# Selections and Abstracts

**Published:** 1917-03

**Authors:** 


### SELECTIONS.

### SYPHILIS AND THE WAR

Dr. H. Vizy makes a rather long article of this in the Journal des Practiciens. He brings out the facts:

That the war has caused a formidable recrudescence of venereal diseases and particularly of syphilis. This was to be foreseen and is now easily demonstrable.

Certainly, during the first months of the fight the troops on the front had small occasion to infect themselves; the hours were too grave and the fatigue too great. But little by little, in the places where, since the war of trenches, the troops go and find a place called "for refreshments," houses of prostitution were opened. In each one of the villages, it may be said that there exists actually two or three women that the troopers pass to one another at the time of "relief." In a few months they become infected with the various venereal diseases. In the cities where the sojourn of the troops is longer and where they have more leisure, prostration flourishes. One daret not speak of the interior zone, which one does not know except by hearsay.

Already reports have been made of the spread of syphilis and especially of the entire families syphilized by a soldier on leave of absence returned to his home for a few days. Neglect or ignorance, the syphilitic in these few days contaminates not only his wife but his children—or the latter are later infected by the mother, generally ignorant of the state in which she is.

It must be known, that if the venereal subject be a bachelor, be sometimes his recourse to the regimental surgeon, the married patient almost never calls upon him. How do these things occur as a fact?

Whereas the inconvenience of gonorrhea may prompt the patient to apply to his regimental surgeon for relief, the initial lesion and the later ones of syphilis are the cause of minor inconvenience, and hence the patient is not driven to the surgeon for relief. With the advice of a nurse or simply by using his own individual package for dressing, the patient treats the primary lesion and does not bother himself with the conquences.

It is necessary to let them know the dangers which they offer to their families and to persuade them to be treated, but at the same time lectures are difficult at the front. They must take place at the depots and be addressed as much to the old as to the young. Soldiers must be shown the danger of infection and the means of protection.

The tracts to be used to best purpose should be brief and bring out boldly three essential points: (1) The dangers of contamination and their prophylaxis; (2) the non-necessity for a man to satisfy an instinct which, however imperious it may be, is never a real need; (3) the need of keeping strength for the struggle, and his health for the race and his family.

The true, perhaps only protection of families is the medical examinations of those about to depart on leave. This examination, which is a regulation, but no longer occurs at the front, should be enforced anew. It is always possible, for it is not indispensable that it take place on the day of departure. It may be made on soldiers designated for a furlough for the week. It should be passed even on the furloughs of short duration and be applied to non-cmmissioned officers as well as privates. It should be individual, complete and scientific.—The Urologic and Cutaneous Review.

---

### EXPERIENCES IN SIBERIA DURING THE WAR, WITH SPECIAL REFERENCE TO THE TYPHUS EPIDEMIC OF 1915.

**By Ethan Flagg Butler, M.D.,**
Yonkers, N. Y.

To better appreciate the factors involved in the outbreak of the epidemic of typhus, and the course thereof, it is advisable to briefly consider the nature of the country and its peoples, and the progress of th war up to that time.

Serbia is hilly, in parts very mountainous. It is divided into a number of river basins, separated from one another by the mountain ranges. Communication along the line of the one railroad, from north to south, and its few branches, is easy; but elsewhere slow and laborious. It was no uncommon thing to be informed, in reply to queries as to distances, that a certain place was so many days railroad, so many days ox-cart, so many days walk, distant. The climate approximates that of southern New York State, or of New Jersey.

It is essentially an agricultural country. To the north are orchards, vineyards, grain fields, and large tracts for grazing. To the south are poppy fields for opium growing, mulberry groves for silk culture, tobacco lands, and pasture lands for sheep and goats. The people, for the most part, are scattered over the hillisdes, or grouped into small, isolated villages, whence they go out to work the surrounding land. There are only a few towns of appreciable size, and but one city worthy of the name, Belgrade, at the northern end of the country, at the junction of the Danube and Sava rivers. Except in the case of Belgrad, which is a well built, modern city, all the towns show, in their architecture, unpaved streets, lack of sewers, and lack of running water, the effect of the Turkish influence which until comparatively recently dominated the land.

Although Serbia has produced some brilliant men, the people, as a whole, are ignorant, slow to learn, and wholly devoid of the least conception of hygiene or sanitation. They are patriotic and brave. Every male member of the population is a soldier, and they are excellent fighters.

In the same way, every physician is a medical officer in the army, either on the active list or on the reserve list. The organization and effciency of the medical department of their army, however, can not be rated very high. It broke down completely under the stress of the typhus epidemic, failing wholly to initiate preventative measures, or to organize any effective care of the sick. There is one excellent military hospital in Belgrad, but no other permanent hospital.

So much for the country. At the outbreak of the great war, Austria launched a strong offensive against Serbia, carrying the fighting onto Serbian soil. A very large proportion of the noncombatant civil population left the zone of the fighting for central and southern Serbia. Overcrowding is these areas naturally resulted, and the sanitary conditions were not such as would safely permit of overcrowding. As the number of injured rapidy inlcreased, Serbia was compelled to call upon foreign medical aid to augment her own medical staff. Reserve hospital points were designated here and there throughout the land, where railroad facilities and available buildings would permit. Patients were rushed to these points even before staffs had been secured to care for the hospitals.

Already the American Red Cross Society had sent one unit, three doctors and twelve nurses, Dr. E. W. Ryan, director, to that country. In answer to the later appeal two more units were organized, and left New York in November, 1914. Dr. E. P. Magruder, of Washington, D. C., was director of Unit 3; Unit 2 was under my charge. The appeal being primarily for surgical units, the personnel was selected and the equipment purchased with that end in view.

Our party reached Serbia when the Austrian drive was at its height. Belgrad, where the first American unit was already located, now fallen into the hands of the enemy and communication was cut off. We could not join forces with Ryan's party and secure, with them, the advantages and security of working within the well appointed Miitlary Hospital. Importuned by the Serbs, the party proceeded to Gevgelia, a primitive little village near the Greek frontier, to take charge of the Reserve Hospital there located.

This hospital proved to be a large warehouse, with great bare lofts and unpartitioned space. There was no running water. There was no adequate system for the disposal of sewage and other waste. Of beds, blankets, mattressts, sheets, there were too few. The kitchen was small and dirty. Laundry facilities were practically nil. Eight hundred and fifty patients, most of them with infected compound fractures, lay within this building. Before we had been there twenty-four hours, 450 more were added.

We had come primarily to render surgical assistance, but we found greater need of sanitation.

There was typhus in Serbia when we reached the country in the middle of December, 1914, but it had not gained the headway to attract more than passing attention. Typhus is endemic in the Balkans, and sporadic cases were constantly occurring. it is impossible to determine where the first cases occurred, but the probabilities are that it started in the central or southern part of the country, in the extempore military hospitals such as the one described, where both Serbs and Austrians were being cared for. As no quarantine measures were enforced, it did not take the disease long to spread to all parts of the land, the spread being especially facilitated by the transfer of the wounded and the Austrian prisoners of war from one part of the country to another. These prisoners were probably a very large factor in the situation, and one of the most active means of the dissemination of the disease. No blame can attach to them, but rather to the methods by which they were handled. As a race they were instinctively clean, but the conditions under which they were housed were by no means ideal, no allowance being made to prevent overcrowding, or to provide the men with the means for bathing or otherwise maintaining their cleanliness. On being taken prisoners they were deprived of all the spare clothing they had. Once louse infested, they had no ready means of freeing themselves of the vermin. With them typhus became a regular thing. There was nothing to indicate that any care was taken, before transferring a squad of prisoners, to delouse them or otherwise safeguard the new post to which they were ordered. Unlike the other countries engaged in the war, Serbia did not keep the prisoners in camps, but distributed them in small groups throughout the land, with very little restraint. In particular, they were freely used as orderlies in the hospitals, where, on account of their superior intelligence and ability, they proved invaluable to all the foreign relief parties operating in that country.

For a time occasional cases were noted, then came a sudden increase in number, and general alarm began to be felt. Early in January, 1915, it was apparent that a serious epidemic existed, and from then until the middle of April, 1915, there was a very rapid increase in the number of cases, and the virulence of the disease. After that it gradually declined.

Our party was in no position to investigate, scientifically, the etiology of the disease. There was nothing in our experience that would tend to contradict the theory that it is louse borne, and only louse borne, and in particular borne by the body of clothing louse. Lice were everywhere; no one, not even of our party, escaped them, though some found more and some less upon themselves. The writer found few lice upon himself, and he was only one of the few who escaped typhus. In support of the louse theory—it is really accepted fact now, and no longer theory—is the proved fact that thorough delousing of the patients prevented the spread of the disease, no cases occurring in the louse free wards, unless they were being incubated at the time of admission, and no ward epidemics existing under those conditions. This we showed at Gevgelia, which became a veritable hotbed of typhus, by keeping one small pavilion entirely free from the disease for two months, while the epidemic was at its height, solely by insisting that no patient should be there admitted unless he were thoroughly deloused. At Belgrad, whither the survivors of our party were later able to go, the same thing was demonstrated. Typhus gained access to the wards of the Military Hospital, early in the course of the epidemic, and a hospital epidemic ensued. By adopting the same routine, and cleaning up the wards one by one, the hospital was freed of the disease, and kept free. Contsant vigilance was essnetial at all times. We could trust none but Americans with the charge of that important feature of the work. In a country such as Serbia, it was much easier to speak of ideal conditions than to attain them.

There was opportunity for us to observe an extremely large number of cases. A census of cases made by the military chief of the hospital at Belgrad showed 922 cases of typhus within the buildings on March 22, 1915. That was the highest figure for any one day. The total number of cases seen must have been well into the thousands. There was also a series of cases occurring among Americans, Serbs, British, Italians and other nationalities, which came under our care. Outside of our own staff invalids, these represented persons of authority that were brought to us that they might secure the benefit of our nursing staff. It would be difficult to bestow too great praise upon the American nurses. This smaller group of cases that we carried as special patients, afforded us an opportunity to minutely follow the course of the disease, and to keep records from which an accurate clinical picture could be drawn. We did not have the available staff members, the time or the laboratory facilities to study in detail the pathological side of typhus.

The incubation period is from seven to fourteen days. In one case it was determined as twelve days. One of the nurses developed a typical lobar pneumonia. On the day of onset, as she was being gotten to bed, lice were found in her clothing. Twelve days later, the pneumonia being over, she came down with typhus. At no other time, after the onset of her pneumonia, had there been lice near her. In no other case could the time be so accurately determined, for lice were found as daily occurrence by the majority of the invalids.

The onset was sudden in almost every case. As a rule the patients had been able to work the day before, but were unable to report on the first day of sickness, or even to get up. In a very few cases the onset was insidious, and masked by symptoms suggesting bronchitis. The symptoms of onset that we noted were, in order of frequency: Severe frontal headache; high fever, 102 degrees to 104 degrees; bronchitis; moderate fever, 100 degrees to 102 degrees; profound prostration; pains in the extremities; parotitis; conjunctivitis. Although we could not make a positive diagnosis of typhus until the skin rash had developed, there were very few cases in which we entertained any doubt as to the ultimate diagnosis for more than one or two days.

In the majority of the cases there was a high temperature throughout the acute course of the disease, two weeks. It either rose at once to the high level or gradually attained it in two or three days. Toward the end of the acute course it would gradually fall, terminating by lysis. The bulk of the text book articles upon typhus state that it terminates by crisis. Crises were noticed in but very few cases, which were marked exceptions to the rule. While elevated, the temperature varied very little, and was not readily lowered by the ordinary methods of bathing, to which we had recourse. Te pulse rate rose with the temperature, and remained rapid throughout. The respirations were not affected unless the degree of bronchitis was marked. There was no sweating. The amount of urnie depended upon the intake of fluids. The bowels, if neglected, moved less frequently than normally.

On the fourth or fifth day there appeared a typical petechial rash, which constituted the surest diagnostic sign of the disease. It appeared on the chest, back, abdomen, extremities and infrequently on the head and face. The individual 'flecks'' were about 2 mm. in diameter, faintly palapable to the examining finger, did not fade on pressure and were of the color of ordinary inflammatory areas of the skin. They increased in number from day to day, the older ones becoming more deeply colored. They did not fade until after the acute course was over, but then disappeared rapidly. As with other acute exanthematica, the severer cases were characterized by a greater number of ''flecks.'' Their size did not change, but where the number was very great they would appear confluent, and lose the discreet appearance. At the time of the appearance of the skin rash, the eyes became very much inflamed, the patients complained of great burning and the vision was impaired.

Throughout the course of the disease there was much pain. At the onset there was the headache, often excruciating. As the days went on pains in the extremities, especially along the tibae, caused great discomfort, until the mental dulling, that eventually ensued, caused the patient to lose track of his sufferings.

As the disease progressed the toxemia became profound. Its effects were to be noted upon the central nervous system, the cardiac muscle and the blood stream and blood vessels. The gastro-intestinal system showed no effects of the disease.

The respiratory system showed only a slight catarrhal inflammation, manifested by the bronchitis already referred to. The urine showed albumen during the acute course, and for the first few days of convalescence, but no other changes.

The disturbances along the central nervous swstem were both motor and sensory, as well as psychic. There was very marked tremor , ataxia, inco-ordination, and involutary passages from bladder and rectum. These latter signs were only noted in the second week, with the disease at its maximum severity. All the senses seemed to be dulled. Phonation was more and more difficult as the course progressed. From the onset there was noted confusion. During the second week disorientation as to all surroundings and time, delusions, illusions and frequently a very active delirium occurred. The patients required a constant watching to prevent them from harming themselves or others, or leaving their quarters. Even so a number of cases comitted suicide during that stage of the disease. With one exception those were general hospital cases.

The heart muscle would show no effect until the latter part of the second week. Then the pulse, that had been regular, rapid, of good volume, and uniform tension, would become irregular in rate and force, and of decreased and varying tension and volume. The heart sounds would be faint and indistinct.

The changes in the blood and the blood vessels would not be apparent, except for the skin rash, until after the cessation of the acute symptoms. Then there would be noted in an appreciable number of patients a more or less extensive gangrene of the extremities, of which there will be more to say.

The acute course lasted from thirteen to seventeen days, with fourteen days in the great bulk of the cases. In one case there was a relapse on the eighth day of convalescence, the patient going through a second typical case of typhus. In that particular instance the patient, a Serbian doctor, had attempted to get up at too early a date.

As the epidemic progressed, and as time went on, the cases became more and more virulent, and the mortality increased.

The early cases among the members of our own staff were not as severe, nor were the squellae as distressing, as the latter cases.

Convalescence was slow at best. The patients were left much weakened, the mentality remained clouded, pains in the extremities persisted. The appetite was voracious, the patients eating anything that was at all edible and within reach. We found it advisable to keep patients lying down for the first week of convalescence, and then to allow them to sit up gradually, it being anotehr week before they would be allowed to leave their beds. In one of the other foreign relief parties, operating in Serbia, deaths were encountered during early convalescence, and attributed by them to acute dilatation of the heart from too early efforts to get the patient up and about. Convalescence was also modified by the sequellae to typhus. These included persistent pains in definite nerve areas, chiefly those of the legs; ataxia continuing for weeks after the cessation of other symptoms; mental disturbances, from slight irritability to definite melancholia, one case of which persisted for at least three months after the acute course; gangrene of toes, feet, legs, thighs, fingers, nose, scrotum, all of which were frequently seen, but were limited without exception to cases in hospitals where very little care could be given the patients. There were also seen huge parotid abscesses, the entire gland lying in the cavities as a free slough; otitis media; mastoid abscesses; and horrible abscesses involving whole extremities, following the fascial planes with masses of muscle lying sloughing in the pus cavities. These types of cases were brought to us from outlying hospitals for the most part. A large percentage died in spite of all efforts to save them. The gangrene cases were extremely unsatisfactory as operative work even though well above the line of demarcation, had to be done in devitalized tissue, tissue that looked more like cooked meat than anything else, and secondary sloughs frequently occurred. Bed sores were often noted. Probably the most potent factor in producing such sequellae to the disease was lack of proper care during the acute course, especially in respect to the amount of water that the patient received. The typical Serbian typhus hospital, of the outlying districts, was not the sort of place in which many patients recovered.

Yet, even with good nursing, unfortunate sequellae occurred in a certain percentage of cases. In the cases that we carried under our own special care, under the very best conditions as to nursing that were attainable in that country, there were noted the following: double parotitis; double purulent otitis media; abscess of submental glands; slough following hypodermoclysis; melancholia, minor psychoses.

What the mortality actually was no one will ever be in a position to say. Such records as were kept were inadequate to express the results, and personally I do not believe that they were in any way accurate. On general services the mortality was not far from 50 per cent., in the acute cases. In addition, many died during convalescence of complications of sequellae, making the net mortality about 60 to 65 per cent. With good nursing, the mortality was much decreased. Of twenty-eight cases, most of them Americans, who received detailed attention in private quarters, and special nursing, only three of 10.7 per cent. died. The cause of death were uremia, suicide, toxemia of typhus.

The staff was not large enough in point of numbers to detail any members to the task of studying the disease scientifically. We could therefore make no contribution to the pathology of typhus, either from autopsy findings, or from studies in the clinical laboratory. That was naturally a source of regret, but was unavoidable.

In making a diagnosis, there had to be excluded relapsing fever, which was also epidemic at the same time, bronchitis, mumps, typhoid. The height of the fever and the degree of the prostration were enough to exclude bronchitis and mumps, even thogh the symptoms of onset might suggest these conditions first. The onset of relapsing fever was ordinarily sudden, with right fever, severe pains in head and other parts of the body, and marked prostration. In typhus, however, the tongue was clearly coated and the conjunctivae inflamed, while the typical rash appeared not later than the fifth day, whereas in relapsing fever the tongue was clean, the conjunctivae clear, there was no rash, the spirochaetae of Obermeyer could be demonstrated in the blood, and the course of the first attack terminated suddenly on the fifth to seventh day. Typhoid could be ruled out by the sudden rise in the temperature, the concomitant increase in the puise rate and the absence of gastro-intestinal symptoms.

The treatment of the disease naturally fell under two heads, prophylactic measures, and the care of the acute cases and their complications and sequellae.

No effective country wide prophylactic work was done prior to the arrival of the foreign sanitary commission from the United States, Great Britain and France. It was extremely difficult for us to secure the enactment of any quarantine orders affecting the hospital within which we were working. Our own active preventative measures were limited to the treatment of the cases within the hospital and the safeguarding of one ward from another. The only satisfactory method was to empty one ward after another of all cases therein, thoroughly clean and fumigate the room, and then return the cases, such as might belong there, after each individual had been completely deloused. Typhus patients, minus lice, were as harmless as yellow fever patients minus mosquitoes. The essential steps in the delousing process consisted of stripping the patients; clipping short all the hary parts of the body; wrapping all material so far removed in a sheet and sending it without delay to the steam sterilizer; giving the patient a cleansing bath of warm water and soap; giving a kerosene shampoo to all hairy parts; furnishing the patient with a new suit of ward clothes. The routine labor of the delousing was carried on by orderlies, but it was necessary to always check the results. The orderlies soon learned, however, that it was better to make one thorough job than to have the same patient come back to them to go through the whole procedure a second time. This routine was slow and arduous in Gevgelia, on account of the lack of running water, and the very poor hospital facilities, but Belgrad it was much easier to put into effective operation. Patients were kept in admitting wards for observation, and if found to be lice free at the end of two or three days, were distributed thence to the wards where they logically belonged. If patients were found by chance to be louse infested, after reaching a louse free ward, they were returned to the admitting rooms, to go through the whole process again, their beds were removed entire, and all other patients in that ward were carefully inspected. If patients developed typhus in one of the surgical or medical wards they were immediately removed to an isolation building for typhus cases, their beds were removed entire, and thoroughly cleansed before being replaced. The doctors and nurses in charge of the medical and surgical wards had nothing to do with the typhus wards, and every effort was made to prevent the Austrian orderlies that were detailed to the typhus wards from gaining access to the typhus free wards. The possibility of these orderlies harboring typhus infected lice was too great to be disregarded.

The routine that we adopted, and found effective, for the acute cases followed closely the type of treatment for typhoid fever in this country. The main dependence was placed upon good nursing, keeping the patient quiet and free from exertion or worry. A fairly liberal soft diet was allowed, and fluids were forced upon the patient. · In every case but two it was easy to get the patient to take by mouth all the fluids necessary. One was the patient lost in uremia, the other patient had a marked melancholia, and resort was had to hypodermoclysis. Toward the end of the second week, when the heart muscle began to weaken, we found various preparations of digitalis of use. The one used most frequently was digilin, hypodermically. Camphorated oil was also occasionally employed. As a rule no drugs were used to quiet the patient in the active delirium. Rarely, to relieve the overburdened nursing staff, in the case of some particularly restless patients, recourse was had to morphine, though it was with great reluctance that we used this measure. The good results that attended our series of special cases was due wholly to the excellent nursing that they received.

The care of the convalescent could not be neglected. Plenty of good food was necessary, and care that the patient did not begin to exert himself too soon. The patient was also apt to be irritable and unreasonble, and in the case of two the mental condition required the presence of a companion at all times.

The complications and sequellae had to be met symptomatically. For the abscesses, drainage was necessary. For the gangrenes, amputation had, of course, to be performed. It was no uncommon thing to have two or three double leg amputations on the morning's surgical list, all the result of typhus. The psychoses slowly cleared up with time.

There was not a relief party in the field that did not pay toll to the typhus. British, French, Greek, Dutch, Rusian (by far the best equipped of any of the parties), American; all suffered depletion in numbers by sickness and death.

Our own first case occurred in the early part of January, 1915· Two more became sick in the latter part of the month. In the early part of February, cases occurred in rapid succession, unti, of the original eighteen, thirteen had come down with the disease. One, Dr. J. F. Donnelly, of New York, died. Dr. E. P. Magruder, of Washington, D. C., was the last member of the party to contract typhus. It was towards the end of March, when we thought that those who had escaped it to date were immune, and would not have it. His case was particularly virulent, and he also died a martyr to the Red Cross work of this war. In all, fourteen out of eighteen in that party had typhus. Of eleven doctors, American and Serbian, in Gevgelia on January 1, 1915, ten had typhus and four of them died. Of the American unit in Belgrad four out of ten, remaining at the time of the epidemic, had the disease. There were no deaths.

They were gruesome days. The filth of the patients as they came in; the close, fetid smell of the wards; the almost endless procession of litter bearers carrying the dead from the hospitals; the rows of dead in the shacks at the outskirts of the town, waiting burial; the dejection of the populace; the new cases occurring among the personel of the staff, the dwindling numbers at the staff dining table; the death of close friends; the funerals of American victims; all these factors made indelible impressions upon us. From time to time uncomfortable thoughts would pass through our minds: "Would I be the next to come down, or worse, would I be the last to come down, after all the other Americans were sick, and none remained to care for me." Frankly, the thought of going through a typhus under Serbian care was far from reassuing. Fotunately, there was so much work to be done, that there was little time for these disquieting thoughts. The spirits of the survivors remained remarkably high. The work of the nurses, throughout, was admirable, and it would be hard to bestow too much praise upon them.

After our Gevgelia party had been decimated by the epidemic, and the convalescents had been started on their way to America, the few survivors, accompanied by relief from other Red Cross Units in Europe, made their way to Belgrad to join the American force there. It seemed almost ironical that the staff epidemic in Belgrad should be just commencing as we reached that city, and that the whole thing had to be gone through with a second time.

Little by little the typhus dwindled, and was already on the decline when the International Sanitary Commission reached the scene. Their work in segregating the remaining cases, and in carrying out a country-wide delousing compaign, with military authority to assist them, completed the work that remained to be done. The disease rapidly disappeared. Freed of cares that the typhus had put upon us, the few of us who remained were able to devote our attention to the surgical tasks that had been accumulating; and to observe, from our hospital on the banks of the Sava, occaisonal brushes between the Ausrupted by the typhus, was gradually resumed.—New York State Medical Journal.

### PRELIMINARY PROGRAM AMERICAN PROCTOLOGIC SOCIETY

Nineteenth annual meeting, New York City, N. Y., June 4th and 5th, 1917. Headquarters and place of meeting, Hotel Astor. The profession is cordially invited to attend all meetings.

### Officers

President, Alfred J. Zobel, M. D., San Francisco, Cal. Vice-President, Granville S. Hanes, M. D., Louisville, Ky., Secretary-Treasurer, Collier F. Martin, M. D., Philadelphia, Pa..

### Executive Council

T. Crittenden Hill, M. D., Chairman, Boston, Mass., Alfred J. Zobel, M. D., San Francisco, Cal.; Wm. M. Beach, M. D., Pittsburg, Pa.; Collier F. Martin, M. D., Philadelphia, Pa.

### PROGRAM

Executive council meets at 8 a. m. First regular session at 9 a. m. Annual address by the President. "The place of the Proctologist in a Diagnostic Group," Alfred J. Zobel, San Francisco, Cal. Memorial address—"Our Late Member, George J. Cook, Indianapolis, Ind., Alois B. Graham, Indianapolis, Ind.

### Papers

Adult Rectal Prolapse; Two Cases and a Contrast, Ralph W. Jackson, Fall River, Mass. Adenomyoma of the Rectum, Frank C. Yeomans, New York City, N. Y. Summary Reports of Nine Cases of Peri-Colic Membrane, John L. Jelks, Memphis, Tenn. Should the Sphincters be Divided? Rollin H. Barnes, St. Louis, Mo. Neglected Rectal Examination, James A. McVeigh, Detroit, Mich. Enemas and Colonic Flushing as Etiologic Factors in Appendicitis, William H. Stauffer, St. Louis, Mo. The Relationship of Hemorrhoidal Disease to the Health Balance, William M. Beach, Pittsburg, Pa. The Underlying Factors of the Clamp and Cautery Operation for Internal Piles, W. Oakley Hermance, Philadelphia, Pa. The

Pathology of Hemorrhoids, J. Coles Brick, Philadelphia, Pa. Report of a Case of Idiosyncracy to Quinine and Urea Hydrochloride, Collier F. Martin, Philadelphia, Pa. Neoproctology.—A Glompse into the Future, Jerome M. Lynch, New York, N. Y. The Post-Operative Factor in Rectal Surgery, Barney J. Dryfuss, New York City, N. Y. The Non-Surgical Treatment of Splanchnoptosis, Rolla Camden, Parkersburg, W. Va.

